# Exploring Whether Making Second-Language Vocabulary Learning Difficult Enhances Retention and Transfer

**DOI:** 10.3390/bs15050692

**Published:** 2025-05-17

**Authors:** Alice F. Healy, Vivian I. Schneider, James A. Kole

**Affiliations:** 1 Department of Psychology and Neuroscience, University of Colorado Boulder, Boulder, CO 80309-0345, USA; vicki.schneider@colorado.edu; 2 School of Psychological Sciences, University of Northern Colorado, Greeley, CO 80639, USA; james.kole@unco.edu

**Keywords:** vocabulary learning, retention, transfer, desirable difficulties

## Abstract

Four previous and two new experiments from our laboratory are reported, in which college students learned associations between French and English words in a learning phase and then took an immediate retention test. One week later, a delayed test was followed by relearning. Four difficulty manipulations were used during learning: blocking versus mixing semantic categories, translation direction, prelearning, and set size. The first new experiment examined the effect of set size on retention, and the second new experiment examined blocking and mixing semantic categories, as well as translation direction, on learning new vocabulary (transfer). Generally, across the six experiments, difficult conditions provided a disadvantage during learning and immediate testing, but made no difference or provided an advantage during relearning and delayed testing. These results suggest that making the initial learning more difficult does not always lead to superior retention.

## 1. Introduction

There are many advantages to possessing a large vocabulary. Studies have shown that knowledge of words’ meanings is crucial for effective communication, enhancing both verbal (e.g., Jongman et al., 2021; Shao et al., 2014; Unsworth et al., 2010) and written (e.g., Olinghouse & Leaird, 2009; Olinghouse & Wilson, 2013) expression. Other studies have shown that vocabulary knowledge is strongly related to reading speed and comprehension (Currie & Muijselaar, 2019; Wagner & Meros, 2010). Moreover, a larger vocabulary contributes to cognitive development, promoting critical thinking and problem-solving abilities, and allowing individuals to engage in more complex reasoning (Kievit et al., 2017, 2019).

Similarly, learning a *second* language also offers numerous cognitive benefits that extend beyond expression and reading comprehension. Bilingualism has been linked to enhanced cognitive flexibility, improved memory, and better multitasking abilities, making bilingual individuals more adept at executive control tasks (Bialystok, 2011; but see Paap et al., 2015, for a different conclusion). Additionally, research suggests that bilingualism may provide neuroprotective effects, potentially delaying the age of onset for symptoms of Alzheimer’s disease (Bialystok, 2021).

Given its importance, many studies have explored methods to improve second language learning, with a strong focus on contextual interference. Contextual interference occurs when multiple skills or variations of a task are interleaved during learning, rather than being practiced in a blocked format (see Battig, 1972, 1979, for the development of the concept of contextual interference). Although this approach leads to slower learning rates, it ultimately enhances both retention and transfer (for a review, see Czyż et al., 2024).

An important early study demonstrating the contextual interference effect was conducted by Shea and Morgan (1979) in the domain of motor skill learning. Subjects learned three distinct movement patterns, each requiring them to knock down three barriers in a specific sequence using their hand. During training, one group practiced in a blocked format, completing multiple trials of one pattern before switching to the next, while another group practiced in a random or interleaved format, alternating between patterns on each trial. Subjects were tested twice: 10 min after training and again 10 days later. The tests included the previously practiced movement patterns (retention), as well as two novel patterns that had not been introduced during training (transfer). The results showed that blocking had different effects on training and test performance. During training, the blocked group performed the movements more quickly than the random group; however, the random group performed better on both the studied patterns and novel patterns during testing, thereby demonstrating better retention and transfer. More recent research has confirmed these findings and expanded on the role of contextual interference in skill acquisition (e.g., Mousavi et al., 2024).

Subsequent research has demonstrated that contextual interference is a more general principle that extends beyond motor learning. For example, in visual category learning tasks for which subjects are shown exemplars of different categories, training might be blocked, such that subjects are presented with exemplars from only one category at a time, or training might be interleaved, such that the exemplars from different categories are intermixed. In general, contextual interference leads to increased retention of the studied exemplars, as well as increased transfer, or the ability to categorize new exemplars (e.g., Carvalho & Goldstone, 2014; Kang & Pashler, 2012; Kornell & Bjork, 2008). Contextual interference has also been shown to improve retention and transfer in more educationally relevant materials, such as chemistry concepts (Eglington & Kang, 2017), and tasks such as classifying statistics problems (Sana et al., 2018) and mathematical problem solving (Mielicki & Wiley, 2022; Rohrer & Taylor, 2007). However, the extent to which contextual interference benefits retention and transfer might vary by task type, with some tasks showing stronger benefits from interleaving than others (Brunmair & Richter, 2019). In certain cases, blocking might enhance retention more than interleaving would (e.g., Carpenter & Mueller, 2013; Sorensen & Woltz, 2016).

Schneider et al. (1998) investigated contextual interference as it relates to second-language vocabulary learning. In two experiments, subjects studied French–English word pairs from five different semantic categories (e.g., body parts or articles of clothing). The study compared two learning conditions: a blocked condition, in which each block of trials during the learning phase included words from only one semantic category, and a mixed condition, in which each block of trials during the learning phase included one word from each semantic category. Subjects in Experiment 1 underwent learning rounds until they responded correctly to all word pairs two times in a row, whereas subjects in Experiment 2 all underwent exactly three learning rounds. Following learning, subjects took an immediate retention test as well as a delayed retention test one week later. Following the delayed retention test, subjects completed three more learning rounds to assess relearning. For both experiments, subjects in the blocked condition demonstrated better learning, but worse relearning after the delay. These findings suggest that although blocking by semantic category might facilitate initial learning, it might not promote long-term retention as effectively as mixing or interleaving semantic categories. However, research on the effects of mixing versus blocking semantic categories during second language vocabulary acquisition has yielded mixed results. Some studies have found that blocking words into semantic categories hinders learning (e.g., Bolger & Zapata, 2011; Tinkham, 1993, 1997), possibly due to the physical similarity between the referents of the words (Bach & Barclay, 2025). In contrast, other studies reported the opposite effect, with blocked presentation leading to better learning outcomes (Hashemi & Gowdasiaei, 2005; Hoshino, 2010).

Contextual interference can be conceptualized as one of a broader class of learning strategies known as *desirable difficulties* (R. A. Bjork, 1994; R. A. Bjork & Bjork, 2020; R. A. Bjork & Kroll, 2015). Desirable difficulties are conditions that introduce challenges during learning, which might initially lead to lower accuracy or a slower learning rate but might ultimately enhance long-term retention and transfer (E. L. Bjork & Bjork, 2011). These difficulties can take many forms, such as the *testing effect* (e.g., E. L. Bjork et al., 2014; for a review, see Yang et al., 2021), in which retrieval practice via testing strengthens memory more than passive restudy, and the *generation effect* (McNamara & Healy, 2000; for a review, see McCurdy et al., 2020), where producing information rather than simply reading it improves recall. Other examples include the *spacing effect* (e.g., Carpenter, 2017), in which learning is distributed over time rather than being massed in a single session, and *variability of practice*, which exposes learners to diverse contexts or problem types (e.g., Raviv et al., 2022).

In a follow-up to their 1998 study, Schneider et al. (2002, Experiment 1) investigated whether contextual interference, as well as the potential desirable difficulty of a hard translation direction, enhances both the retention and transfer of second language vocabulary items. Second language vocabulary learning is inherently bi-directional, requiring two complementary translation processes: one from the second language to the first and the other in the reverse direction. Experiment 1 examined both translation processes and the transfer between them. Specifically, half of the subjects (all of whom did not speak French) completed a learning phase and an immediate retention test using French words as cues and English words as responses, representing a less difficult translation task. The remaining subjects completed a learning phase and an immediate retention test with English words as cues and French words as responses, a more challenging translation task. All subjects completed three learning rounds before the immediate retention test.

In a second session, conducted one week after learning, subjects completed a delayed retention test followed by relearning. During relearning, they either continued with the same translation direction as in the learning phase or switched to the opposite direction. To examine all possible combinations of first and second tasks, four groups were employed: (a) French-to-English first, French-to-English second; (b) English-to-French first, English-to-French second; (c) French-to-English first, English-to-French second; and (d) English-to-French first, French-to-English second. A contextual interference manipulation was also included, as in Schneider et al.’s (1998) study, with items either blocked by semantic category or mixed. Thus, each group was further divided into four subgroups based on the practice schedules used across the two sessions: (e) blocked first, blocked second; (f) mixed first, mixed second; (g) blocked first, mixed second; and (h) mixed first, blocked second.

As predicted, the manipulation of translation direction had the opposite effects on learning and relearning, similar to the earlier demonstrations of contextual interference. Specifically, accuracy was much higher during learning for the French-to-English translation direction than for the English-to-French translation direction. This advantage was also evident in the immediate test. However, subjects given the more difficult English-to-French translation direction during learning showed higher accuracy on the delayed retention test one week later, as well as somewhat higher accuracy during relearning, regardless of the translation direction used during relearning. The latter result demonstrates that increasing the difficulty of the task during learning aids in the long-term retention of the original task, as well as in transfer to a new task.

Other studies obtained similar results regarding translation direction and retention. Griffin and Harley (1996) found that presenting first-language cues and requiring second-language responses (L1-L2) during learning, as in the English-to-French translation direction of Schneider et al. (2002), resulted in greater retention on comprehension and production tests than the easier L2-L1 (second language cues, first language responses) translation direction, possibly due to the increased retrieval demands and deeper processing. Others, including Terai et al. (2021), revealed a similar pattern, but only when subjects were more proficient with the second language. Webb (2009) found that L1-L2 translation direction increased retention as well as orthographic and syntactic knowledge.

Schneider et al. (2002, Experiment 2) introduced a second manipulation focused on learning difficulty (in this case *reducing* it) by providing prelearning for half of the French words. For prelearning, subjects were presented with a French word and simply had to type it. Each prelearned word appeared three times across three prelearning rounds. Following prelearning, as in Experiment 1, subjects completed a learning phase and an immediate retention test, as well as a delayed retention test and a relearning phase during a second session one week later. Also, as in Experiment 1, half of the subjects were assigned the more difficult English-to-French translation direction, whereas the other half were assigned the less difficult French-to-English direction. In the second session, half of the subjects in each condition continued with the same translation direction, whereas the other half switched to the opposite direction for both the delayed retention test and relearning. For Experiment 2 there was not a blocked/mixed manipulation; for all subjects, the words were mixed across semantic categories.

As in Experiment 1, subjects in the French-to-English translation condition performed better during learning than those in the English-to-French translation condition. However, during relearning in Session 2, those in the English-to-French condition showed greater improvement across sessions, despite initially performing worse. Similarly, subjects assigned to the easier French-to-English condition during learning outperformed those in the English-to-French condition on the immediate test, but this advantage slightly reversed on the delayed retention test.

A comparable pattern emerged for prelearned versus non-prelearned words. During learning in Session 1, subjects performed better on prelearned words than on non-prelearned words. However, by Session 2, the performance improvements were greater for non-prelearned words than for prelearned words during relearning. Despite these differences in learning, prelearning had no effect on test performance.

Another difficulty manipulation explored in both first- and second-language vocabulary learning is block size, or the number of words studied together before testing. In a study of first-language vocabulary, Kornell (2009) found that studying all vocabulary items in a single block led to better retention than dividing them into four smaller blocks. Nakata and Webb (2016) investigated block size in second-language learning and found that larger blocks led to a reduced performance during learning rounds. However, block size only had a consistent effect on final test performance when controlling for spacing, or the interval between the repeated presentations of a word or block. Similarly, Nakata et al. (2023) reported that increasing spacing during learning enhances retention, and that this benefit becomes even more pronounced when spacing is repeated during relearning phases.

In summary, contextual interference occurs when learning tasks are interleaved rather than blocked. As demonstrated in early studies of motor learning (Shea & Morgan, 1979), this effect was subsequently observed in category learning (e.g., Carvalho & Goldstone, 2014; Kang & Pashler, 2012; Kornell & Bjork, 2008), chemistry (Eglington & Kang, 2017), mathematics (Mielicki & Wiley, 2022; Rohrer & Taylor, 2007), statistical problem-solving (Sana et al., 2018), and the acquisition and retention of logic rules (Schneider et al., 1995). Contextual interference is just one of many desirable difficulties that lead to slower initial learning but enhance retention and transfer. In previous studies by (two of) the present authors (Schneider et al., 1998, 2002), both contextual interference and desirable difficulties were examined in a second-language vocabulary learning task. These studies showed that increasing difficulty, whether through contextual interference, translation direction, or a prelearning manipulation, results in better performance during learning, but reduced performance in a delayed test or during relearning.

The present study follows Schneider et al. (1998, 2002) by providing two new experiments that examine other manipulations of task difficulty and their effects on learning, relearning, retention, and transfer on a task involving learning a second language.

Unlike earlier studies by others, using varied methodologies and often yielding contradictory results, we use the same general methodology in these new experiments as in our earlier studies, enabling us to examine vocabulary learning, relearning, retention, and transfer in a consistent, controlled way across different task-difficulty manipulations.

## 2. Experiment 1

In Experiment 1, to manipulate difficulty, we varied the block size, or the number of words that were presented together during learning. Previous studies have also examined this issue. Kornell (2009) compared block sizes of 4 and 20, whereas Nakata and Webb (2016) compared block sizes of 4, 10, and 20. In the present experiment, subjects either completed the learning phase with a smaller set size, where each block included only six words, or with a larger set size, where each block included twelve words. Experiment 1 focused on retention rather than transfer, with subjects completing an immediate test and a delayed test (as in Schneider et al., 1998, 2002), as well as a relearning phase. Increasing the block set size might create a desirable difficulty, such that retention is enhanced. However, increasing difficulty might also result in cognitive overload, which impairs retention and transfer (Xie et al., 2017).

### 2.1. Method

#### 2.1.1. Subjects

A total of 48 University of Colorado undergraduates participated as subjects for credit in an introductory psychology course. All of these subjects were native English speakers who did not speak French. Previous studies examining block size and retention (e.g., Kornell, 2009; Nakata & Webb, 2016), typically yielded large effect sizes. A power analysis based on Kornell (2009; η^2^ = 0.21) suggests that 32 subjects would be sufficient to achieve 0.8 power for the main effect of set size in the present experiment. An analysis based on Nakata and Webb (2016, Experiment 2; η^2^ = 0.41) indicates that as few as 14 subjects are needed to achieve 0.8 power. The difficulty manipulation (prelearning) used by Schneider et al. (2002; η^2^ = 0.25) suggests that 26 subjects would be adequate to achieve sufficient power. These studies varied in terms of factors such as whether they examined first- or second-language vocabulary learning, the difficulty manipulation used, the language learned, and the set size.

#### 2.1.2. Materials

Thirty-six French words were used in this experiment, including six words from each of six different semantic categories: body parts, school, transportation, dining, food, and clothing. (For a list of the 36 items used in this study, see Appendix A).

#### 2.1.3. Procedure

Experiment 1 included two sessions that were spaced one week apart. The first session included a learning phase and an immediate retention test; the second session included a delayed retention test followed by a relearning phase.

For both the learning and relearning phases, in each trial, subjects were presented with a French word and its English translation for a period of 2 s. In the *small set size* condition, one word was presented from each of six categories, and after six French word–English translation pairs were presented, subjects were tested over the preceding six items. Subjects were provided with the French word and had to type the corresponding English translation. This learning/test cycle repeated until the 36 words were presented and tested three times. The *large set size* condition proceeded in a similar manner. However, subjects in this condition were tested after 12 French word–English translation pairs were presented, rather than 6, and the 12 pairs included two words from each of six categories. During learning, half of the subjects were assigned to the small set size condition and the other half to the large set size condition; during relearning, half of the subjects remained in the same condition they were in during learning, whereas the other half switched to the other set size condition.

For both the immediate and delayed retention tests, subjects were tested with all 36 words via cued recall, as occurred during the learning rounds. More specifically, they were tested in the French-to-English direction over all 36 items without further study.

#### 2.1.4. Design

For the analyses of learning phases, the design was a 2 × 2 × 2 × 3 mixed factorial. There were two between-subjects variables: set size for the learning phase (large, small) and set size during relearning. There were also two within-subjects variables: session (Session 1 and Session 2) and learning round (Round 1, Round 2, and Round 3).

For the analysis of the test, the design was a simpler 2 × 2 × 2 mixed factorial including the variables of set size during learning (large, small), set size during relearning (large, small), and test (immediate, delayed).

The dependent variable for both learning and testing was the proportion of correct responses.

### 2.2. Results

For both Experiments 1 and 2, we analyzed our results using analyses of variance (ANOVAs).

#### 2.2.1. Learning

**Figure 1:**
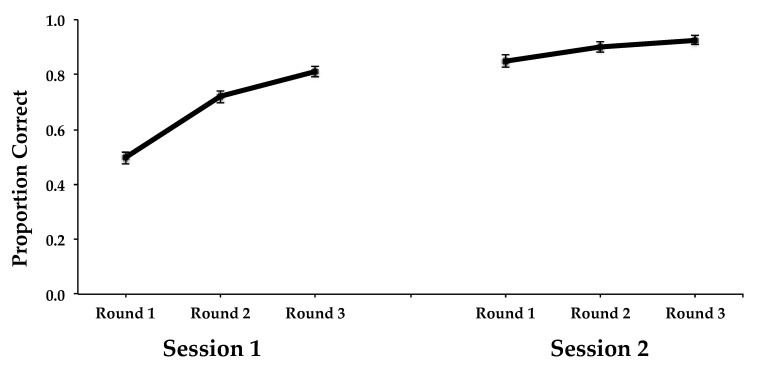
Proportion of correct answers during learning and relearning in Experiment 1 as a function of learning round (Round 1, Round 2, Round 3) and session (Session 1, Session 2). Error bars represent between-subjects standard errors of the mean.

**Figure 2:**
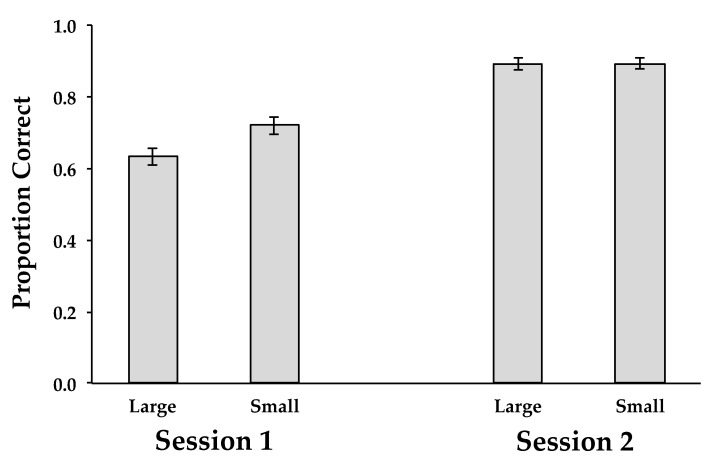
Proportion of correct answers during learning and relearning in Experiment 1 as a function of learning set size (Large, Small) and session (Session 1, Session 2). Error bars represent between-subjects standard errors of the mean.

For learning, there was a main effect of session (*F*(1, 44) = 236.720, *MSE* = 0.014, *p* < 0.0001, and η^2^ = 0.843), reflecting the learning from the first (*M* = 0.675) to the second (*M* = 0.891) session. Learning was also reflected in the significant main effect of learning round (*F*(2, 88) = 257.418, *MSE* = 0.004, *p* < 0.0001, and η^2^ = 0.854), with an increase from the first round (*M* = 0.673) to the second round (*M* = 0.809), and to the third round (*M* = 0.868). There was also a significant interaction between session and learning round (*F*(2, 88) = 107.872, *MSE* = 0.003, *p* < 0.0001, and η^2^ = 0.710), reflecting the greater improvement across rounds observed in the first session compared to the second (see Figure 1). Most importantly, there was also an interaction between learning set size and session, (*F*(1, 44) = 9.639, *MSE* = 0.014, *p* < 0.0001), and η^2^ = 0.180. Specifically, overall accuracy was higher for subjects in the small learning set size condition than for those in the large learning set size condition in Session 1, but was virtually the same in Session 2 (see Figure 2). Thus, the improvement across sessions was greater for the large learning set size condition than for small learning set size condition.

#### 2.2.2. Test

The only significant effect during testing was the main effect of session, *F*(1, 44) = 165.150, *MSE* = 0.005, *p* < 0.0001, η^2^ = 0.790, reflecting words that were forgotten between the immediate test (*M* = 0.727) and the delayed test (*M* = 0.541).

### 2.3. Summary and Discussion

Experiment 1 manipulated difficulty by varying the set size or the number of items that were presented and tested at a time. As with other studies investigating desirable difficulties, increasing difficulty had different effects on learning and retention over time. Initially, those in the small learning set size condition performed better, but by the second session, accuracy was similar across learning set size conditions. This finding suggests that the large learning set size condition, despite being more difficult initially, did not impair relearning. This finding is also interesting given the research on procedural reinstatement, which states that retention and transfer are maximized when the learning and testing procedures match (Healy et al., 1992). According to procedural reinstatement, those who were in the same set size condition during learning and relearning should have performed better than those who switched. However, there was no advantage during relearning for those in the same condition as they were in during learning. There were also no differences between the learning set size conditions regarding the test results, which might suggest that there is no advantage in terms of retention of using larger set sizes, although we did not control for spacing (Nakata & Webb, 2016). However, as research on the testing effect has shown, successful retrieval during learning might not impact test performance (e.g., Chan et al., 2024).

## 3. Experiment 2

Experiment 2 explored the extent to which transfer is affected by increasing task difficulty. Until this point, our studies on transfer were limited to variations in the translation direction (Schneider et al., 2002). An examination of transfer from one list of word pairs to a completely different list is equally important. In Experiment 2, we investigated this type of transfer. Subjects learned words from six semantic categories during the first session; during the second session, subjects learned new words from the same six semantic categories, as well as new words from different semantic categories. The learning rates and memory for the two types of words were compared to determine whether previous experience with the semantic categories facilitates the learning of new words from those categories. Generally, research shows that prior knowledge can facilitate the learning of domain-related information (e.g., Kole & Healy, 2007; Van Overschelde & Healy, 2001), whereas specific studies of second-language vocabulary learning show that blocking words into semantic categories can hinder learning (e.g., Bolger & Zapata, 2011; Tinkham, 1993, 1997). However, the present study focuses on transfer to the learning of new items.

### 3.1. Method

#### 3.1.1. Subjects

Again we employed a sample of 48 University of Colorado undergraduate students participating as subjects for credit in an introductory psychology course. All of these subjects were native English speakers who did not speak French. Power estimates were based on studies by Tinkham (1993, 1997) because they were methodologically similar to the present experiment. Specifically, vocabulary items were presented as word pairs, and the primary dependent variable was a measure of accuracy. For these studies, the η^2^ for the main effect of relatedness (grouping semantic items vs. unrelated items) was large (Tinkham, 1993, η^2^ = 0.59; Tinkham, 1997, Experiment 1, η^2^ = 0.51, and Experiment 2, η^2^ = 0.37). These effect sizes suggest that between 8 and 16 subjects would be sufficient to achieve 0.8 power. However, the focus of the present study is on whether or not semantic categories influence the learning of new items rather than on how well the items in the semantic categories are learned relative to semantically unrelated items.

#### 3.1.2. Materials

The materials were 96 French–English word pairs (12 words from each of eight semantic categories). (For a list of the 96 items used in this study, see Appendix B).

#### 3.1.3. Procedure

For the first session, subjects completed the learning phase, where they learned 36 word pairs; the words were presented in a mixed format, with one word from each of the six semantic categories in each block. As in Experiment 1, each French word/English translation was presented for 2 s, and after six French words/English translations were presented, subjects were tested using cued recall. Subjects completed three learning rounds, with each of the 36 word pairs being presented and tested three times. Subjects were given an immediate test of all 36 word pairs. For the purpose of analysis, these 36 word pairs were classified into three categories, SW, SC, and DC, based on the counterbalancing scheme. Across subjects, counterbalancing ensured that word pairs were assigned equally often to each of these categories.

For the second session, which was held one week later, subjects completed a delayed test, followed by three rounds of learning, and then a final test. Subjects again learned and were tested over a total of 36 words. However, only 12 words were the same as those in the learning phase. Those 12 words included 6 words from each of two of the six categories employed during Session 1 (SW; same words). Another 12 words were 6 new words from each of another two categories employed in Session 1 (SC; same category), and the third set of 12 words consisted of 6 new words from each of two new categories that were not previously employed during the learning phase (DC; different category). Thus, the breakdown of word pairs into categories was meaningful only in the second session.

In the first week, half of the subjects learned and were tested in a French-to-English translation direction, and the remaining half learned and were tested in an English-to-French translation direction. For the second week all subjects were tested and relearned in the harder English-to-French direction.

#### 3.1.4. Design

The design for the analysis of learning performance was a 2 × 2 × 3 × 3 mixed factorial, with translation direction during learning (English-to-French or French-to-English), as the between-subjects variable, session (Session 1 or Session 2), learning round (Round 1, Round 2, or Round 3), and word type (SW words used in both sessions, SC words used only during relearning from previously studied categories, or DC words used only during relearning from unstudied categories) as the within-subject variables.

The design of the analysis of test performance was a 2 × 3 × 3 mixed factorial, with translation direction during learning in Week 1 (English-to-French or French-to-English) as the between-subjects variable, and test (immediate, delayed, or final), and word type (SW, SC, or DC) as the within-subject variables.

The dependent measure during the learning rounds and the tests in each of the two sessions was the proportion of correct responses.

### 3.2. Results

#### 3.2.1. Learning

**Figure 3:**
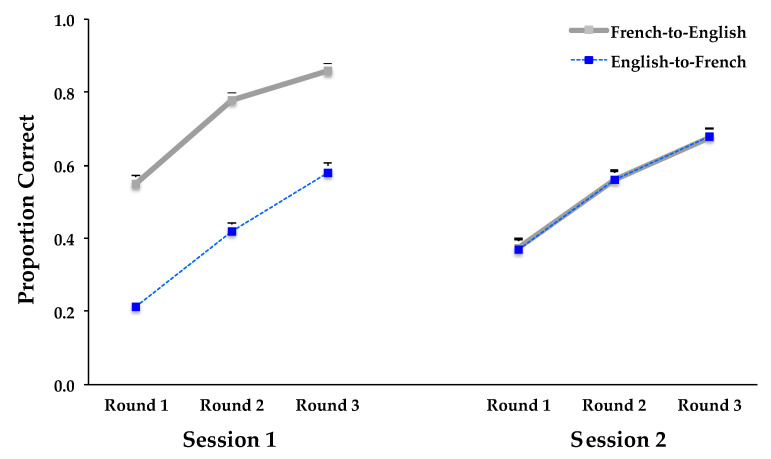
Proportion of correct answers during learning and relearning in Experiment 2 as a function of learning translation direction (French-to-English, English-to-French), session (Session 1, Session 2), and learning round (Round 1, Round 2, Round 3). Error bars represent the between-subjects standard errors of the mean.

Overall performance during learning in Session 1 was much better for subjects learning in the French-to-English direction than for those given the English-to-French direction; however, there was no difference between the two groups during relearning in Session 2 (see Figure 3). Thus, there was an interaction between translation direction during learning and session, *F*(1, 46) = 117.369, *MSE* = 0.049, *p* < 0.0001, and η^2^ = 0.718, as well as a main effect of translation direction during learning, *F*(1, 46) =19.433, *MSE* = 0.295, *p* < 0.0001, and η^2^ = 0.297, and a main effect of session, *F*(1, 46) = 4.218, *MSE* = 0.049, *p* = 0.0457, and η^2^ = 0.084.

Both the French-to-English and the English-to-French groups improved across learning rounds in both sessions; however, during relearning, when both groups performed the more difficult task of translating from English to French, the English-to-French group improved greatly from Session 1 whereas the French-to-English group declined in accuracy from Session 1 (see Figure 3). The interaction of translation direction, session, and learning round was significant (*F*(2, 92) = 3.787, *MSE* = 0.007, *p* = 0.0263, and η^2^ = 0.076), as was the interaction of session and learning round (*F*(2, 92) = 3.727, *MSE* = 0.007, *p* = 0.0278, and η^2^ = 0.075), and the main effect of learning round (*F*(2, 92) = 396.290, *MSE* = 0.019, *p* < 0.0001, and η^2^ = 0.896).

**Figure 4:**
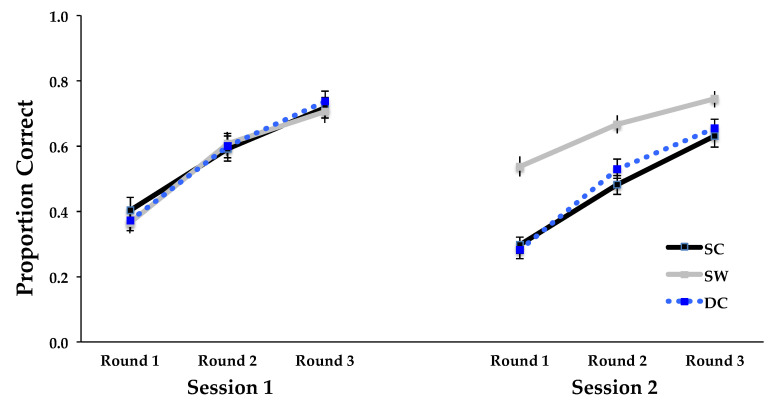
Proportion of correct answers during learning and relearning in Experiment 2 as a function of word type (different categories, DC; same words, SW; and same categories, SC), session (Session 1, Session 2), and learning round (Round 1, Round 2, Round 3). Error bars represent between-subjects standard errors of the mean.

A better performance was obtained during relearning in Session 2 for the SW word type, or words that were included in both learning and relearning phases, than for either set of other words only shown during relearning (SC, DC), with the advantage for the SW word type decreasing across relearning rounds (see Figure 4). The interaction between session, learning round, and word type was significant (*F*(4, 184) = 4.960, *MSE* = 0.011, *p* = 0.0008, and η^2^ = 0.097), as was the main effect of word type (*F*(2, 92) = 9.536, *MSE* = 0.065, *p* = 0.0002, and η^2^ = 0.172). The component interactions between word type and session (*F*(2, 92) = 32.628, *MSE* = 0.024, *p* < 0.0001, and η^2^ = 0.415), and between word type and learning round (*F*(4, 184) = 6.350, *MSE* = 0.010, *p* < 0.0001, and η^2^ = 0.121) were also significant.

To examine transfer, an analysis was conducted that was restricted to the three blocks of relearning during Session 2, and included only the SC and DC categories. Both SC and DC words were new during Session 2; however, subjects learned words from the same semantic categories as SC words during Session 1, but had no previous exposure to the semantic categories for DC words. This analysis revealed no significant main effects or interactions involving the word type variable. However, because subjects were tested over only six word pairs at a time during relearning blocks, the differences between these word types might have been minimized.

#### 3.2.2. Test

**Figure 5:**
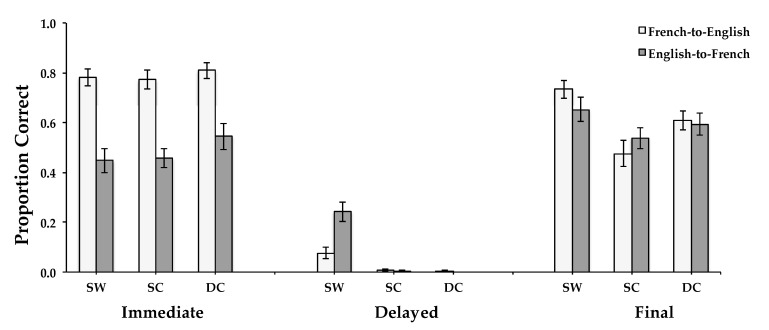
Proportion of correct test answers in Experiment 2 as a function of learning translation direction (French-to-English, English-to-French), test (Immediate, Delayed, Final), and word type (different categories, DC; same words, SW; and same categories, SC). Error bars represent between-subjects standard errors of the mean.

Overall, accuracy was higher for those who learned using the easier French-to-English direction than those who learned using the more difficult English-to-French direction; the main effect of translation direction during learning was significant: *F*(1, 46) = 6.557, *MSE* = 0.124, *p* = 0.0138, and η^2^ = 0.125 (see Figure 5). Accuracy was also higher for the immediate and final tests than for the delayed test; the main effect of the test was significant: *F*(2, 92) = 482.105, *MSE* = 0.032, *p* < 0.0001, and η^2^ = 0.913. The main effect of word type was significant as well, as, on average, across tests, accuracy was highest for SW words, lowest for SC words, and intermediate for DC words: *F*(2, 92) = 16.415, *MSE* = 0.028, *p* < 0.0001, and η^2^ = 0.263.

The interaction between translation direction, test, and word type was significant: *F*(4, 184) = 7.329, *MSE* = 0.013, *p* < 0.0001, and η^2^ = 0.137. For the immediate test, there was a more consistent advantage across word types for the French-to-English direction than for the English-to-French direction. However, for the delayed test, performance was better for the harder English-to-French direction than for the easier French-to-English direction for SW words, which were included during the learning session. Performance was worst for both types of new words (SC and DC, both of which included words that were not presented during the learning phase). For the final test, neither translation direction was consistently superior (see Figure 5). The component interactions between test and translation direction (*F*(2, 92) = 41.227, *MSE* = 0.032, *p* < 0.0001, and η^2^ = 0.473), and between test and word type (*F*(4, 184) = 15.483, *MSE* = 0.013, *p* < 0.0001, and η^2^ = 0.252), were also significant.

To test for transfer, a planned comparison between the SC and DC word types at the final test was conducted. The difference between word types was significant (*F*(1, 46) = 13.587, *MSE* = 0.020, *p* = 0.0006, and η^2^ = 0.228) with a higher accuracy for the DC than for the SC word types (see Figure 5). Thus, negative transfer (or interference) occurred, as previous experience with semantic categories hindered memory of new words from those categories.

### 3.3. Summary and Discussion

Experiment 2 examined the effects of both translation direction difficulty and blocking words into semantic categories during second-language vocabulary learning. Previous studies have investigated blocking by semantic category (e.g., Bolger & Zapata, 2011; Tinkham, 1993, 1997), with the general finding that such blocking can impair retention. The present study also tested for transfer by examining learning and memory for new words belonging to previously studied categories, as well as unstudied categories.

Similarly to the study by Schneider et al. (2002; Experiment 1), the more difficult English-to-French translation direction resulted in lower accuracy during both the learning and the immediate test than the French-to-English translation direction; however, these differences were virtually eliminated during relearning. Thus, increasing the learning difficulty during the first session did not result in a superior performance during the second, although it did not decrease performance. Thus, at least according to the findings of this experiment, translation direction does not appear to be a desirable difficulty for second-language vocabulary learning.

Subjects demonstrated significant forgetting from the immediate test to the delayed test. However, there was some evidence of retention in that words that were presented during both sessions (SW) were learned more quickly than new words introduced during Session 2 (SC, DC). In terms of transfer, there was a clear *negative* transfer effect: during the final test, subjects recalled words from entirely new categories (DC) at a higher rate than new words from previously studied categories (SC). Although this result is somewhat surprising given previous research that knowledge of a domain makes learning domain-relevant information more efficient (e.g., Kole & Healy, 2007), second-language vocabulary learning studies consistently yielded the opposite pattern, presumably due to interference (e.g., Tinkham, 1993, 1997).

## 4. General Discussion

The present study investigated whether making second-language vocabulary learning more difficult enhances retention and transfer, extending the framework of desirable difficulties (R. A. Bjork, 1994; R. A. Bjork & Bjork, 2020; R. A. Bjork & Kroll, 2015) and contextual interference (e.g., Battig, 1972, 1979; Czyż et al., 2024). Across two experiments, we examined the effects of two difficulty manipulations, including set size during learning (Experiment 1), as well as translation direction and blocking according to semantic category (Experiment 2), on second-language vocabulary acquisition, retention, relearning, and transfer.

Experiment 2 further explored transfer by assessing how the semantic relatedness between learning and relearning materials influences new learning. Previous studies (e.g., Tinkham, 1993, 1997) found that learning words in semantic clusters impairs the retention of those words compared to mixing words from different semantic categories. Experiment 2 examined transfer by assessing whether knowledge of exemplars from a semantic category negatively impacts the learning of new members of that category. Indeed, this pattern was observed in Experiment 2: subjects performed worse on words from previously studied categories (SC) than on words from entirely new categories (DC) during the final test, indicating a negative transfer effect. This result suggests that prior exposure to semantically related content can produce interference rather than facilitation, consistent with earlier findings in the second-language-learning literature (e.g., Tinkham, 1993, 1997).

Thus, a consistent pattern that emerged across both experiments was that manipulations that increased difficulty during learning (i.e., a larger set size in Experiment 1 and English-to-French translation direction in Experiment 2) were associated with reduced performance during learning. These effects were large; however, the disadvantage for more difficult learning conditions was eliminated during relearning and the final test in each experiment. These findings are partially consistent with the prior research on desirable difficulties, which generally show a reduced performance during learning but superior performance on delayed testing. Together, these findings show that not all manipulations that introduce difficulty appear to produce long-lasting benefits, or long-lasting harm.

There are limitations to the present study. As mentioned previously, Experiment 1 did not control for spacing, and it is possible that larger set sizes could improve retention if spacing was controlled. If so, then block size could be considered a desirable difficulty when learning second-language vocabulary, as long as spacing is controlled. In Experiment 2, a delayed retention test was not administered for the SC and DC items; thus, it is possible that the SC items, which were more difficult to learn during the second session, may have ultimately been better retained. Regardless of the type of difficulty manipulation, future research should investigate how difficulty influences other aspects of learning. For example, increasing difficulty is presumably perceived as requiring increased effort, and increased effort can be perceived as an indication of lesser learning (de Bruin, 2023) and can also result in decreased motivation.

## Data Availability

Data are available upon request from the authors.

## Appendix A. Items Used in Experiment 1

*Category*	*French*	*English*
body parts	jambe	leg
	dos	back
	bouche	mouth
	figure	face
	yeux	eyes
	doigt	finger
clothing	manteau	coat
	chemise	shirt
	chaussures	shoes
	cravate	tie
	jupe	skirt
	lunettes	glasses
dining	fourchette	fork
	cuiller	spoon
	couteau	knife
	assiette	plate
	verre	glass
	serviette	napkin
food	pain	bread
	beurre	butter
	lait	milk
	oeufs	eggs
	jambon	ham
	fromage	cheese
school	cahier	notebook
	livre	book
	stylo	pen
	ordinateur	computer
	devoir	assignment
	note	grade
transportation	voiture	car
	velo	bicycle
	avion	airplane
	moto	motorcycle
	camion	truck
	bateau	boat

## Appendix B. Items Used in Experiment 2

*Category*	*French*	*English*
body parts	bouche	mouth
	bras	arm
	coeur	heart
	cou	neck
	doigt	finger
	dos	back
	genou	knee
	jambe	leg
	main	hand
	yeux	eyes
	oreille	ear
	figure	face
clothing	calecon	shorts
	chapeau	hat
	chaussures	shoes
	chemise	shirt
	gant	glove
	lunettes	glasses
	manteau	coat
	robe	dress
	cravate	tie
	veston	jacket
	jupe	skirt
	tailleur	suit
dining	jatte	bowl
	assiette	plate
	couteau	knife
	nappe	tablecloth
	tasse	cup
	fourchette	fork
	cuiller	spoon
	verre	glass
	louche	ladle
	serviette	napkin
	timbale	mug
	cruche	pitcher
food	aubergine	eggplant
	beurre	butter
	epices	spices
	farine	flour
	fromage	cheese
	gateau	cake
	haricots	beans
	legume	vegetable
	nouille	noodle
	oeufs	eggs
	pain	bread
	cerise	cherry
people	epoux	spouse
	fille	girl
	fils	son
	garcon	boy
	homme	man
	soeur	sister
	boulanger	baker
	ecrivain	writer
	femme	women
	pretre	priest
	reine	queen
	roi	king
places	bibliotheque	library
	ecole	school
	eglise	church
	epicerie	grocery
	magasin	store
	maison	house
	piscine	pool
	plage	beach
	gare	station
	ferme	farm
	immeuble	apartment
	salle	room
school	valise	bag
	cahier	notebook
	livre	book
	note	grade
	ordinateur	computer
	stylo	pen
	craie	chalk
	crayon	pencil
	devoir	assignment
	pupitre	desk
	chaise	chair
	grattoir	eraser
transportation	bateau	boat
	camion	truck
	chaloupe	canoe
	fusee	rocket
	velo	bicycle
	velomoteur	moped
	voiture	car
	cheval	horse
	planeur	glider
	avion	plane
	charrette	wagon
	patin	skate
